# Investigating Socioeconomic Disparities in Access to and Utilization of Assisted Reproductive Technology Among Infertile Couples in Pakistan: A Cross‐Sectional Study

**DOI:** 10.1002/hsr2.71603

**Published:** 2025-12-02

**Authors:** Muhammad Shaheer Bin Faheem, Hafiza Qurat Ul Ain, Syed Tawassul Hassan, Faheem Feroze, Wajeeha Imam, Ayesha Khalid, Qasra Faheem, Sumaya Samadi

**Affiliations:** ^1^ Karachi Institute of Medical Sciences, KIMS Karachi Pakistan; ^2^ CMH Multan Institute of Medical Sciences Multan Pakistan; ^3^ Karachi Medical and Dental College, KMDC Karachi Pakistan; ^4^ Combined Military Hospital Rawalpindi Rawalpindi Pakistan; ^5^ Nishtar Hospital Multan Pakistan; ^6^ Kabul University of Medical Sciences “Abu Ali Ibn Sina” Kabul Afghanistan

**Keywords:** assisted reproductive techniques, infertile couples, infertility, Pakistan, socioeconomic disparities

## Abstract

**Background:**

The socioeconomic inequalities play a major role in determining the access and use of assisted reproductive technology (ART) by infertile couples in low‐ and middle‐income nations. Our goal was to identify and compare the sociodemographic variables related to the access and use of ART between the couples who use it and those who do not in Pakistan.

**Methods:**

The comparative cross‐sectional study was conducted at the infertility clinic of CMH Multan between January 1, 2024 and June 30, 2024. Women (aged 18–45 years) were consecutively recruited to the ART group (*n* = 150) and the non‐ART group (*n* = 150). A structured questionnaire was used in gathering sociodemographic data, medical history, and treatment information. The *t*‐tests and *χ* ^2^ tests were used to conduct descriptive and bivariate analyses. The variables with *p* 0.05 were put into a multivariate binary logistic regression model to determine independent predictors of ART use.

**Results:**

Women in the ART group were older (mean age 31.25 ± 4.69 years) and had longer infertility periods, were better educated and had better incomes, were more likely to be employed, urban dwellers, and had nuclear families. Multivariate analysis has revealed that higher education, urban residence, nuclear family, prolonged infertility, and ART awareness were independent predictors of ART use (*p* < 0.05). The most prevalent barrier (46%) to treatment access was the presence of financial constraints.

**Conclusions:**

The socioeconomic status is a key determinant of ART uptake in Pakistan. The affordability, the expansion of ART services provided by the public sector, and the spreading of awareness are needed to achieve fair access to infertility care.

## 1 Introduction

Infertility is defined as the inability to conceive after 1 year of regular unprotected intercourse. This issue affects 8%–12% of couples worldwide, with prevalence rates rising up to 16.7% in low‐middle‐income countries (LMICs) [1, 2]. In Pakistan, around 22% of the population experiences infertility, and the country's cultural context largely links this issue exclusively with the marital identity and childlessness of a woman who then faces social stigma, psychological distress, and marital instability, while male factors or inexplicable circumstances are frequently overlooked [3, 4, 5].

Assisted reproductive technologies (ARTs) such as in vitro fertilization (IVF), intracytoplasmic sperm injection (ICSI), and intrauterine insemination (IUI) are a group of treatments that aim to achieve conception through the handling of gametes or embryos, transforming the infertility treatment globally [6]. However, these services are predominantly offered at private health centers in Pakistan and remain out of reach for lower‐income groups [3, 5, 7]. The typical price of one IVF cycle ranges from about PKR 250,000–500,000 and may reach up to PKR 700,000 based on the clinic and procedures. For instance, ICSI, which is often added on top of standard IVF, can cost an additional PKR 40,000–70,000, while even the relatively simpler IUI treatment costs around PKR 45,000–60,000. This cost burden is a significant financial hit and restriction to ART utilization, especially in a country where infertility care is mostly privately funded [8, 9, 10].

India faces comparable problems as the majority of fertility facilities operate privately, insurance coverage is limited, and treatment expenses are high enough to push the idea of parenting out of reach for low‐income or rural couples [11]. In spite of the aforementioned, the weight of cultural stigma in both Pakistan and India enforces people turning to traditional healers instead of doctors, or hesitating to seek help, underlining a problem that extends beyond money [12, 13]. Lack of education, poor awareness, and the utter shortage of ART clinics outside major cities intensify the gaps on both sides of the border [12, 13]. In contrast, Iran has tried to integrate infertility care into its public health framework more actively through its policies. Yet people run into familiar barriers, such as treatment costs, deep‐seated cultural sensitivities, and the urban–rural divide in healthcare access [14, 15]. A similar structural divide was observed at the global level in high‐income countries, where higher education, income, and private‐sector reliance predict greater ART use, while limited public coverage and private clinic concentration maintain disparities [16, 17, 18, 19].

In contrast, Iran has tried to integrate infertility care into its public health framework more actively through its policies. Yet people run into familiar barriers, such as treatment costs, deep‐seated cultural sensitivities, and the urban–rural divide in healthcare access [14, 15]. A similar structural divide was observed at the global level in high‐income countries, where higher education, income, and private‐sector reliance predict greater ART use, while limited public coverage and private clinic concentration maintain disparities [16, 17, 18, 19].

However, there is a few evidence from South Asian LMICs, particularly in Pakistan, where cultural expectations, economic pressures, and poor health infrastructure are different from higher‐income countries and significantly influence ART access and utilization. Given this gap, we aim to determine these socioeconomic determinants and compare them between ART users/intended users and nonusers at a Pakistani tertiary infertility clinic. Our findings will inform Pakistani healthcare policy on ART and reproductive health.

## 2 Methods

This comparative cross‐sectional study was carried out at CMH, Multan Infertility Clinic, between January 1, 2024 and June 30, 2024.

### 2.1 Participants and Sampling

A total of 320 infertile women (defined as inability to conceive after ≥ 12 months of regular unprotected intercourse) were recruited, and only those were classified in ART group (*n* = 150) who had initiated or completed any ART procedure or those who had formally decided or registered to initiate ART within the study period while 150 received no ART. Consecutive sampling was used to include all the eligible participants and to minimize selection bias. Women with a known medical condition contraindicated ART treatment or who were not willing to give an interview were excluded from the study. Among 320 eligible women who were approached, 20 (6.25%) refused to participate mainly because of privacy concerns. No additional information was obtained with these nonrespondents.

### 2.2 Recruitment and Data Collection

Trained medical officers approached the eligible patients during routine clinic visits and interviewed them using a self‐designed structured questionnaire after taking informed consent. The questionnaire was designed on the basis of evidence across studies and localized to sociocultural context covering (i) sociodemographic characteristics (age, education, employment, family structure, household income, residence); (ii) infertility history (type, duration, previous treatments); (iii) awareness and sources of information about ART; and (iv) barriers to ART utilization. Further patients' records were utilized for the medical history and treatment details.

### 2.3 Ethical Approval

The Ethical Committee and Review Board of CMH Multan provided approval for this study under File No. 13/Trg (ERC No. 62/2024, dated January 2024), and a written informed consent was obtained from all participants, and data confidentiality was assured.

### 2.4 Sample Size Calculation

An expected ART utilization prevalence of 10.29% was used to calculate the estimated sample size (*n* = 142) with 5% precision and a 95% confidence interval (CI) [20]. However, a total of 150 participants were included in each group to increase the statistical power of this study.

### 2.5 Statistical Analysis

Data were analyzed using SPSS version 25. Continuous variables were reported as means with standard deviations, while categorical variables were presented as frequencies and percentages. *χ* ^2^ tests for categorical variables and independent *t*‐tests for continuous variables were applied to find the significance of these variables. All the tests were two‐sided, while significance was kept at *p* < 0.05. Significant variables (*p* < 0.05) in bivariate analyses were included in a multivariate binary logistic regression analysis to identify significant independent predictors of ART usage (coded 1 as users/intended users and 0 as nonusers). Adjusted odds ratios (aOR) and 95% CI were used. Variable Inflation Factors (VIF) were used to calculate multicollinearity between the predictors; the tolerance values exceeded 0.7, and VIF values were below 1.5, representing no significant multicollinearity (Table S1). SPSS was used to conduct the analyses (V 26). The study strictly adheres to the STROBE guidelines for cross‐sectional studies, and the SAMPL statements were followed for the reporting of statistical methods [21, 22].

## 3 Results

The mean age of participants in this study was 31.25 ± 4.69 years, with an age range of 23–44 years. Women enrolled in the ART group (31.9 ± 4.9 years) were slightly older and had longer marriage durations (6.5 ± 2.6 years) than those without ART (age: 30.7 ± 4.4 years; marriage duration: 5.4 ± 2.1 years).

### 3.1 Sociodemographic Characteristics

Women who received or intended to use ART had significantly higher levels of education, with 17.3% having completed graduation or above, compared to 6% in nonusers. Employment rates were significantly doubled in ART users (28% vs. 16%). Only 3.3% of the nonusers reported a monthly household income of PKR > 100,000 compared to 13.3% in the ART group, and the majority (70%) had incomes below PKR 50,000. The majority of the ART population resides in Urban areas (65.3%), having more frequent nuclear family systems (67.3%) compared to higher rural residence rates (49.3%) and extended family systems (45.3%) in non‐ART users (Table 1).

**Table 1:** Comparison of demographics and socioeconomic data.

*n* = 300				
Sociodemographic factors		ART group (*n* = 150)	Non‐ART group (*n* = 150)	*p*
Age (mean ± SD) years		31.86 ± 4.89	30.65 ± 4.4	*p* = 0.02
Time since marriage (mean ± SD) years		6.46 ± 2.6	5.4 ± 2.11	*p* < 0.001
Educational level	Graduation or above *n* (%)	26 (17.3)	9 (6)	*p* = 0.01
	Secondary to H secondary *n* (%)	68 (45.33)	70 (46.6)	
	Middle or below *n* (%)	56 (37.3)	71 (47.3)	
Employment status	Employed *n* (%)	42 (28)	24 (16)	*p* = 0.01
	Household *n* (%)	93 (62)	97 (64.6)	
	Unemployed *n* (%)	15 (10)	29 (19.3)	
Monthly household income	> 100,000 Rs *n* (%)	20 (13.3)	5 (3.3)	*p* < 0.001
	50,001–100,000 Rs *n* (%)	46 (30.6)	38 (25.3)	
	< 50,000 Rs *n* (%)	84 (56)	107 (71.3)	
Residential status	Urban *n* (%)	98 (65.3)	76 (50.6)	*p* = 0.01
	Rural *n* (%)	52 (34.6)	74 (49.3)	
Family system	Nuclear *n* (%)	101 (67.3)	82 (54.6)	*p* = 0.025
	Extended *n* (%)	49 (32.6)	68 (45.3)	

### 3.2 Infertility Characteristics

Data regarding infertility type showed that the majority of the couples were suffering from primary infertility in both groups (ART: 91.3% vs. non‐ART: 94.6%). However, significantly longer infertility durations were observed in the ART group (6.2 ± 2.3 years) compared to nonusers (5.3 ± 1.9 years). Almost half of the participants reported previous infertility treatments in both groups, while ART‐related awareness was significantly higher in users (68% vs. 45%) (Table 2).

**Table 2:** History related to infertility.

*n* = 300				
Information related to infertility		ART group (*n* = 150)	Non‐ART group (*n* = 150	*p*
Type of infertility	Primary *n* (%)	137 (91.3)	142 (94.6)	*p* = 0.37
	Secondary *n* (%)	13 (8.6)	8 (5.3)	
Previous infertility treatments *n* (%)		82 (54.6)	70 (46.6)	*p* = 0.82
Duration of infertility (mean ± SD) years		6.16 ± 2.29	5.27 ± 1.86	*p* < 0.001
Awareness about ART *n* (%)		102 (68)	68 (45.3)	*p* < 0.001

### 3.3 Utilization and Barriers to ART

IVF was the most commonly used therapy in the ART group, followed by IUI (30%) and ICSI (24%). Doctors were the primary source of ART information (42.6%), followed by social media (17.3%) and family/friends (5.3%), while the majority of those in the non‐ART group had no reliable information regarding ART (54.6%). Financial constraints were the top barrier in access and utilization of ART among nonusers, followed by lack of awareness (40%) and cultural or religious beliefs (9.3%) (Table 3).

**Table 3:** Information and utilization of ART treatment.

*n* = 300			
Information and utilization of ART treatment		ART group (*n* = 150)	Non‐ART group (*n* = 150)
Source of information	Doctor *n* (%)	64 (42.6)	28 (18.6)
	Social media *n* (%)	26 (17.3)	21 (14)
	Family/friends *n* (%)	8 (5.3)	15 (10)
	Others *n* (%)	4 (2.6)	4 (2.6)
	No information *n* (%)	48 (32)	82 (54.6)
Type of infertility treatment	IVF *n* (%)	67 (44.6)	N/A
	IUI *n* (%)	45 (30)	N/A
	ICSI *n* (%)	36 (24)	N/A
	Others *n* (%)	2 (1.3)	N/A
Barriers in accessing ART	Financial constraints	N/A	69 (46)
	Lack of awareness	N/A	60 (40)
	Cultural/religious belief	N/A	14 (9.3)
	Others	N/A	7 (4.6)

### 3.4 Multivariable Analysis

The multivariate binary logistic regression model explains 26.1% of ART utilization variance (Nagelkerke *R* ^2^ = 0.261) and is statistically significant (*χ* ^2^ = 65.30, df = 11, *p* < 0.001). The model correctly classified 69.7% of cases (Table S2). Following the adjustment, a range of sociodemographic factors remained significant independent predictors of ART use. Graduate women or those with higher education were nearly four times more likely to use ART (AOR = 3.80, 95% CI: 1.46–9.89, *p* = 0.006), while earning a high income had fivefold higher odds (AOR = 5.14, 95% CI: 1.46–18.04, *p* = 0.011). Moreover, urban residents (AOR = 2.25, *p* = 0.003), employed women (AOR = 2.70, *p* = 0.036), and nuclear families all used ART more frequently. Each year of infertility increased the odds by 30% (AOR = 1.31, *p* = 0.014). ART use doubled with awareness (AOR = 2.00, *p* = 0.011) (Table 4).

**Table 4:** Multivariable binary logistic regression of factors associated with ART utilization.

Variables in the equation									
		*B*	S.E.	Wald	df	Sig.	Exp(B)	95% CI for EXP(B)	
								Lower	Upper
Step 1^a^	Participant's age (years)	−0.028	0.045	0.383	1	0.536	0.972	0.890	1.063
	Participant's highest education level			9.992	2	0.007			
	Participant's highest education level (1)	−0.339	0.421	0.651	1	0.420	0.712	0.312	1.625
	Participant's highest education level (2)	1.334	0.489	7.449	1	0.006	3.795	1.456	9.891
	Monthly household income category			6.926	2	0.031			
	Monthly household income category (1)	0.669	0.437	2.348	1	0.125	1.953	0.830	4.595
	Monthly household income category (2)	1.637	0.641	6.523	1	0.011	5.138	1.463	18.040
	Place of residence (urban/rural) (1)	0.813	0.277	8.635	1	0.003	2.254	1.311	3.876
	Employment status (yes/no)			4.416	2	0.110			
	Employment status (yes/no) (1)	0.539	0.389	1.922	1	0.166	1.714	0.800	3.672
	Employment status (yes/no) (2)	0.993	0.472	4.416	1	0.036	2.699	1.069	6.812
	Type of family system (nuclear/extended) (1)	0.594	0.276	4.639	1	0.031	1.812	1.055	3.112
	Duration of infertility (years)	0.266	0.108	6.024	1	0.014	1.305	1.055	1.614
	Awareness of ART (yes/no) (1)	0.692	0.274	6.401	1	0.011	1.998	1.169	3.415
	Constant	−2.727	1.190	5.250	1	0.022	0.065		

*Note: B*, coefficient; S.E., standard error; Wald, test statistic; Sig, *p* value; Exp(B), odds ratio; 95% CI for Exp(B).

^a^ Variable(s) entered on Step 1: Participant's age (years), participant's highest education level, monthly household income category, place of residence (urban/rural), employment status (yes/no), type of family system (nuclear/extended), duration of infertility (years), awareness of ART (yes/no).

## 4 Discussion

We examined how socioeconomic inequality affects ART access and use in infertile couples in Pakistan. We found that women who utilized or planned to receive ART have higher education and employment rates, belong to higher income households, and urban areas with nuclear family structures. Further, these women were aware of the treatment options and relied more on doctors as their primary information source. Conversely, financial barriers were the major reason for inaccessibility to the ART treatment, which was further dominated by a lack of awareness and cultural and religious beliefs. Multivariable analysis confirmed that socioeconomic factors independently influence ART use. Higher education, income, employment, and urban residence were associated with ART use, as were nuclear families, longer infertility duration, and awareness. These findings show that material and educational benefits strongly influence reproductive care access in Pakistan.

Similar trends have been observed in other LMICs, such as India, where these reproductive treatments are frequently confined to urban private sector clinics, excluding the rural and low‐income populations due to high cost and limited availability of fertility clinics [11]. Further, there is a widespread cultural stigma in India and Pakistan, especially in rural areas, that supports the reliance on spiritual healers and attributes infertility to supernatural causes, which further restricts the timely diagnosis and treatment. Over 98.7% of tribal women in India prefer traditional healers, while in Pakistan, 28% of the adults consider alternative treatment options over doctors, with strong beliefs in evil forces and spiritual causes, which are considered a common coping strategy to manage the emotional burden of infertility [12, 13]. However, Iran has favorable reproductive health policies, but ART is only available to those couples who meet the religious demands, while the high associated cost and limited clinics further make it inaccessible to the low‐income groups [14, 15]. The study conducted in Senegal also supports our results by demonstrating the significance of cost burden and awareness as determining factors of ART use, particularly within a resource‐restrained environment [23].

The Pakistani society is largely patriarchal and pronatalist, and the causes of infertility are mostly blamed on the female, with no reference to any medical, male, or unexplained factors. They are also blamed for their childlessness, particularly when expectations of the extended family system with regard to the bearing of children are not achieved [13, 24]. Consequently, women suffer serious psychosocial, social, and physical effects such as abuse, exclusion, stigma, threat of divorce, and pressure on their husbands to remarry [13, 24].

In addition, the outrageous price associated with ART is a significant financial burden on the rural or poor couple since the cost of one round of IVF treatment falls between PKR 550,000 and 6,50000, and ICSI (PKR 40,000–70,000) and IUI (PKR 45,000–60,000) are supplementary additions [8, 9, 10]. Such direct cost makes this treatment unaffordable to most infertile couples in Pakistan, where fertility services are hardly insurable and the role of the public sector is practically nonexistent [8, 9, 10]. In addition, ART care is centralized in big cities of Pakistan like Karachi and Lahore, and restricted to the private clinics, while the rural people who form the majority of the population in the country have little or no access [25]. These results demonstrate that inequalities in the treatment of ART in Pakistan are not only determined by the economic variations but also by the deep‐rooted sociocultural beliefs and require comprehensive policies that can be undertaken to mitigate them at the health system and policy levels, particularly in LMICs.

Comprehensive approaches are needed to address these inequities at the health system and policy levels, especially in LMICs. In 2012, the national health system of Brazil recognized ART as a universal right, making these services available at no direct cost to patients. However, most LMICs lack such types of public funding, national subsidy programs, and insurance coverage for ART that are essential to ease the financial burden for couples with low income [26]. Low‐cost ART protocols such as mild ovarian stimulation and projects like Walking Egg and INVOcell aim to adapt ART for low‐income settings and can lower the direct IVF cost but have not been scaled or evaluated widely for their safety and efficacy in LMICs [5].

ART services must be expanded beyond large metropolitan hospitals to primary care and community‐based sites to address geographic disparities, as it increases rural access and reduces travel costs for patients [26]. Further public awareness campaigns and mobile counselling services should be launched to increase knowledge regarding infertility and its treatment, which will eliminate the misconceptions that put false blame on women and have shown to improve infertility‐related stigma and psychological distress [26, 27]. Primary and secondary healthcare providers should be instructed to identify and make referrals to infertility, which will enhance the timely access and eliminate workload gaps in rural communities [26]. Finally, the role of religious and social norms should not be overlooked as they might help to tailor the interventions accordingly with the encouragement of evidence‐based practices [13]. Taken together, all these measures can potentially uplift the ART access and use throughout Pakistan and could serve as an example of the LMICs with similar challenges.

This study has a number of limitations. It was carried out at one tertiary infertility clinic and might not be applicable to the rest of the regions in the country, which differ in accessibility and culture. This recruitment can also be associated with selection bias since women who are in need or want to be in need of ART are probably different from those who do not visit fertility centers. Although multivariable logistic regression was performed to address the possible confounders, unmeasured variables and residual confounding cannot be eliminated, and the model described a moderate degree of variance. A cross‐sectional design does not allow causal inference. The questionnaire was self‐designed and had not been pilot tested or psychometrically validated, and the data collected were self‐reported and might be subject to recall or social desirability bias. Not all eligible participants agreed to the interview, and no information on them was available, limiting the ability to evaluate the bias of responses. Lastly, even though there was a measurement of information sources, there was no measurement of the accuracy of the knowledge of the respondents. The research gives valuable preliminaries to understanding socioeconomic disparities in ART access in Pakistan despite these limitations.

## 5 Conclusion

The results of our study confirm that socioeconomic status, such as levels of education, household income, residential location near or far from the medical facility, family system, and level of awareness, significantly contribute to disparities in access to and utilization of ART treatment among infertile couples. Hence, financial constraints play a major role in this disparity, followed by a lack of awareness and cultural/religious beliefs.

## Author Contributions

**Muhammad Shaheer Bin Faheem:** conceptualization, methodology, software, validation, formal analysis, investigation, data curation, writing – original draft, writing – review and editing, visualization, supervision, and project administration. **Hafiza Qurat Ul Ain:** conceptualization, methodology, resources, data curation, and visualization. **Syed Tawassul Hassan:** investigation, validation, data curation, writing – original draft, writing – review and editing, resources, and project administration. **Faheem Feroze:** investigation, validation, data curation, writing – original draft, writing – review and editing, resources, and project administration. **Wajeeha Imam:** software and validation. **Ayesha Khalid:** investigation, resources, and data curation. **Qasra Faheem:** data curation, resources, and project administration. **Sumaya Samadi:** data curation, resources, and project administration. All authors have read and approved the final version of the manuscript.

## Disclosure

The Muhammad Shaheer Bin Faheem affirms that this manuscript is an honest, accurate, and transparent account of the study being reported; that no important aspects of the study have been omitted; and that any discrepancies from the study as planned (and, if relevant, registered) have been explained.

## Ethics Statement

The Ethical Committee and Review Board of CMH Multan provided approval for this study under File No. 13/Trg (ERC No. 62/2024, dated January 2024).

## Consent

Written informed consent was obtained from all participants, and data confidentiality was assured.

## Conflicts of Interest

The authors declare no conflicts of interest.

## Supporting information


**Table S1:** Multicollinearity diagnostics for independent variables [Variance Inflation Factor (VIF) and Tolerance]. **Table S2:** Model performance indicators (a: Model Summary, b: Classification Accuracy, c: Hosmer–Lemeshow test).

## Data Availability

The data that support the findings of this study are available from the corresponding author upon reasonable request. Sumaya Samadi had full access to all of the data in this study and take complete responsibility for the integrity of the data and the accuracy of the data analysis.
