# Supplementary material for: Investigating Socioeconomic Disparities in Access to and Utilization of Assisted Reproductive Technology Among Infertile Couples in Pakistan: A Cross‐Sectional Study
Source: Health Sci Rep. 2025 Dec 2;8(12):e71603. doi: 10.1002/hsr2.71603 (PMC12672917; doi:10.1002/hsr2.71603)
Supplement: Supplementary file 1 — Table S1: Multicollinearity diagnostics for independent variables [Variance Inflation Factor (VIF) and Tolerance]. Table S2: Model performance indicators (a: Model Summary, b: Classification Accuracy, c: Hosmer–Lemeshow test). [file HSR2-8-e71603-s001.docx]

| Variable | Tolerance | VIF |
| --- | --- | --- |
| Participant’s highest education level | 0.807 | 1.239 |
| Monthly household income category | 0.772 | 1.295 |
| Place of residence (Urban/Rural) | 0.933 | 1.071 |
| Employment status (Yes/No) | 0.874 | 1.145 |
| Type of family system (Nuclear/Extended) | 0.959 | 1.043 |
| Duration of infertility (years) | 0.913 | 1.095 |
| Awareness of ART (Yes/No) | 0.9 | 1.111 |

**Table S1.** Multicollinearity diagnostics for independent variables [Variance Inflation Factor (VIF) and Tolerance]

**Table S2.** Model performance indicators (a: Model Summary, b: Classification Accuracy, c: Hosmer–Lemeshow Test).

1. **Model Summary**

| **Model Summary** | | | |
| --- | --- | --- | --- |
| Step | -2 Log likelihood | Cox & Snell R Square | Nagelkerke R Square |
| 1 | 350.593^a^ | .196 | .261 |
| a. Estimation terminated at iteration number 5 because parameter estimates changed by less than .001. | | | |

1. **Classification Accuracy**

| **Classification Table^a^** | | | | | |
| --- | --- | --- | --- | --- | --- |
|  | Observed | | Predicted | | |
|  |  |  | Utilization or intention to use ART (User/Non-user) | | Percentage Correct |
|  |  |  | No | Yes |  |
| Step 1 | Utilization or intention to use ART (User/Non-user) | No | 104 | 46 | 69.3 |
|  |  | Yes | 45 | 105 | 70.0 |
|  | Overall Percentage | |  |  | 69.7 |
| a. The cut value is .500 | | | | | |

1. **Hosmer–Lemeshow Test**

| **Contingency Table for Hosmer and Lemeshow Test** | | | | | | |
| --- | --- | --- | --- | --- | --- | --- |
|  | | Utilization or intention to use ART (User/Non-user) = No | | Utilization or intention to use ART (User/Non-user) = Yes | | Total |
|  |  | Observed | Expected | Observed | Expected |  |
| Step 1 | 1 | 27 | 25.937 | 3 | 4.063 | 30 |
|  | 2 | 25 | 22.528 | 5 | 7.472 | 30 |
|  | 3 | 18 | 20.188 | 12 | 9.812 | 30 |
|  | 4 | 19 | 18.006 | 11 | 11.994 | 30 |
|  | 5 | 15 | 16.018 | 15 | 13.982 | 30 |
|  | 6 | 9 | 13.990 | 21 | 16.010 | 30 |
|  | 7 | 13 | 12.266 | 17 | 17.734 | 30 |
|  | 8 | 11 | 10.431 | 20 | 20.569 | 31 |
|  | 9 | 10 | 7.582 | 21 | 23.418 | 31 |
|  | 10 | 3 | 3.053 | 25 | 24.947 | 28 |
